# Knee Impedance Modulation to Control an Active Orthosis Using Insole Sensors

**DOI:** 10.3390/s17122751

**Published:** 2017-11-28

**Authors:** Ana Cecilia Villa-Parra, Denis Delisle-Rodriguez, Jessica Souza Lima, Anselmo Frizera-Neto, Teodiano Bastos

**Affiliations:** 1 Postgraduate Program in Electrical Engineering, Federal University of Espirito Santo, Vitoria 29075-910, Brazil; delisle05@gmail.com (D.D.-R.); frizera@ieee.org (A.F.-N.); 2 Biomedical Engineering Research Group GIIB, Universidad Politécnica Salesiana, Cuenca 010105, Ecuador; 3 Center of Medical Biophysics, University of Oriente, Santiago de Cuba 90500, Cuba; 4 Postgraduate Program in Biotechnology, Universidade Federal do Espirito Santo, Vitoria 29043-900, Brazil; jpaola.fisio@gmail.com

**Keywords:** active knee orthosis, admitance control, footswitch, gait cycle, knee impedance

## Abstract

Robotic devices for rehabilitation and gait assistance have greatly advanced with the objective of improving both the mobility and quality of life of people with motion impairments. To encourage active participation of the user, the use of admittance control strategy is one of the most appropriate approaches, which requires methods for online adjustment of impedance components. Such approach is cited by the literature as a challenge to guaranteeing a suitable dynamic performance. This work proposes a method for online knee impedance modulation, which generates variable gains through the gait cycle according to the users’ anthropometric data and gait sub-phases recognized with footswitch signals. This approach was evaluated in an active knee orthosis with three variable gain patterns to obtain a suitable condition to implement a stance controller: two different gain patterns to support the knee in stance phase, and a third pattern for gait without knee support. The knee angle and torque were measured during the experimental protocol to compare both temporospatial parameters and kinematics data with other studies of gait with knee exoskeletons. The users rated scores related to their satisfaction with both the device and controller through QUEST questionnaires. Experimental results showed that the admittance controller proposed here offered knee support in 50% of the gait cycle, and the walking speed was not significantly different between the three gain patterns (*p* = 0.067). A positive effect of the controller on users regarding safety during gait was found with a score of 4 in a scale of 5. Therefore, the approach demonstrates good performance to adjust impedance components providing knee support in stance phase.

## 1. Introduction

Walking is more difficult for persons that suffer gait impairments due to age, stroke, paralysis or spinal cord injury [1,2]. They usually present muscles weakness, knee instability, gait asymmetry and reduction of gait velocity [3,4], which may produce alterations in sensory or motor systems, leading to injury, disability, risk of falls, loss of independence and reduction in the quality of life [5].

Robotic assisted systems can provide functional compensation for lower-limbs during gait, making possible to improve the human locomotion assistance and the gait rehabilitation through powered exoskeletons and active orthoses [6,7,8,9]. The objective of these devices is to help lower-limb impaired people to make their joints move through external movement compensation, using suitable mechanical structures, actuators and control systems. Preliminary findings report promising results, as the fact of sub-acute stroke patients experimenting added benefit from exoskeletal gait training [10], and powered exoskeletons providing individuals with thoracic-level motor-complete spinal cord injury the ability to walk [11].

For the implementation of proper gait training and rehabilitation plans, control strategies that consider both the ability and impairment of the user are required [12]. In this sense, an impedance controller offers the possibility of regulating the mechanical impedance at joints according to the user’s disability level and their voluntary participation to promote a compliant human–robot interaction [13,14,15]. Here, the impedance is regulated through the relation between force, position and its time-derivate, which is given by three components: stiffness, damping and inertia. Thus, a robotic-assisted system can provide interactive gait training adjusting the amount of support to be assisted [8]. In fact, some reviews report that the use of an adaptive impedance control strategy provides a gait motion training that is comparable to the one provided by physical therapists [6].

In this context, some robotic devices use variable impedance, such as the mechanism reported in [16], which employs a variable damping to substitute the stabilizing effect of eccentric quadriceps’ contractions during stance flexion in walking. In addition, there is the robotic orthosis reported in [14], which uses an adaptive impedance control to provide assistance at low compliance level to severely impaired subjects adapting the compliance to an increased level for subjects with less severe impairments. In [17], an impedance control is used as method to effectively transfer the task-oriented impedance profile from the human master to the robotic slave device.

Due to the fact that humans change their joint impedances during gait by regulating the postures and the muscle-contraction levels to maintain the stability, robotic devices must integrate methods for a suitable impedance modulation to assist the movement through the gait cycle. Impedance modulation allows promoting a compliant human-robot interaction to provide an effective human support through assisting the limited motor capability of the user [12,13]. Despite this, few studies have explored suitable and reliable methods to execute this modulation in gait applications, which are necessary in rehabilitation robots to guarantee a dynamic performance [18]. The literature provides information about impedance modulation for assist-as-needed control strategies based on interaction torque estimation methods and trajectory references [8,12,18,19]. In this case, a common limitation is the discontinuous model, just like to turn on or off the robotic assistance, rather than offering a seamless impedance tuning process [18]. Robots that use manual impedance level adjustment to adapt the support to patient’s capabilities or training progress have also been reported [12]. Some methods try to estimate the joint stiffness using electromyography signals combined with kinetic and kinematic measurements to estimate muscle force, together with models that relate muscle force to stiffness [20,21], which would be of great interest for control strategies. However, these methods have still not been applied in control systems for robotic devices to assist gait.

Furthermore, strategies such as stance control (SC) using impedance control have been little explored, although it is reported as a strategy that can be used to increase walking speed, reduce energy expenditure and gait asymmetry (for both affected and unaffected legs), allowing less stress for paretic musculature in patients with muscular weakness [22,23,24]. A stance control strategy provides knee stability and protects the joint from collapsing during the standing and stance phase of walking, releasing the knee to allow free motion during the swing phase [25].

A study about the mechanics of the knee during the stance phase of the gait, reported in [26], suggests that, ideally, the mechanism that adjusts impedance at the knee should be based on the gait speed and weight in order to mimic the behavior of the human knee joint. In this sense, a suitable impedance modulation can allow a smooth switching between the stance phase and swing phase to apply impedance compensation during gait under the SC principle, which is a remarkable challenge to warranty a suitable response of robotic orthosis [25,27]. Thus, in contrast with mechanical knee orthoses, a stance controller implemented with a variable impedance controller can be considered as a promising orthotic intervention for assistive devices, in order to provide the patient with adequate knee stability and allow a more normal gait.

The objective of this work is to propose a new method for online impedance modulation to switch the knee impedance throughout the gait cycle in order to implement a stance controller with an admittance controller (one of the variations of impedance controllers). Our impedance modulation method uses the gait velocity, height and weight of the user to generate a gain variable pattern to increase or decrease impedance parameters during gait. Information about the gait phases obtained from an instrumented insole composed of force sensors is used. To validate the approach, the controller was implemented and tested, with different subjects, in an active knee orthosis.

The novelty of the proposed control scheme relies on the use of the footswitch data of the instrumented insole to regulate the knee impedance of the user without additional sensors, through the generation of gain patterns that adjust the impedance components. Previous studies show that instrumented insoles provide information about plantar pressure that can be used to implement strategies for human motion recognition [28] and detect gait sub-phases [29]. In addition, due to the fact that it is a method based on direct measurement of ground reactions having high accuracy [30], several analyses of walking strategies in stroke survivors and older adults are being developed based on data gathered from these instrumented insoles [31,32].

The method proposed here uses two gain variable patterns, which are based on knee torque and knee velocity during gait, in order to evaluate the suitable condition to implement the SC controller. This control strategy also provides the possibility of investigating knee impedance variations in humans, such as done by other studies focused on upper limbs [13], which is of vital interest to researchers involved with the design and control of variable impedance prosthetic and orthotic devices [33]. This paper is organized as follows. Section 2 presents the admittance control strategy and the knee impedance adjustment method, the gait phase detection system composed of an instrumented insole, the description of the active knee orthosis and the experimental protocol used to validate the controller. Section 3 shows experimental results to evaluate the method, and Section 4 presents the discussion.

## 2. Materials and Methods

### 2.1. Admittance Controller

**Figure 1:**
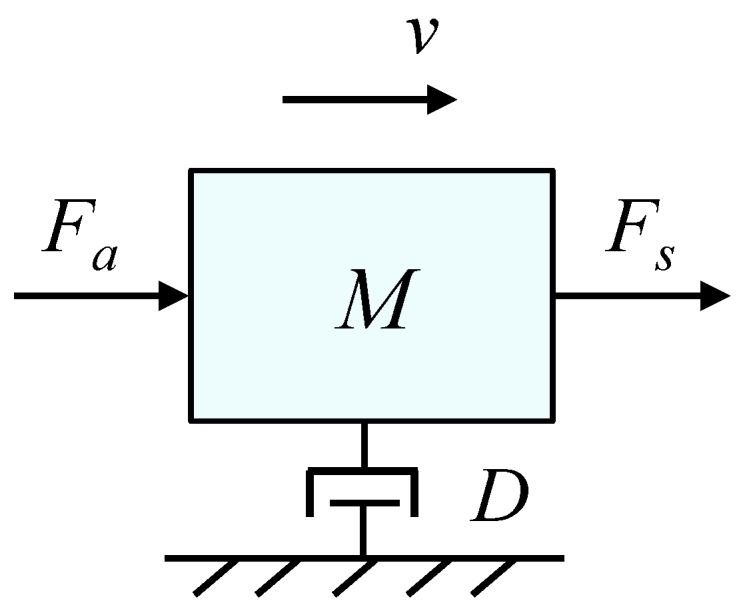
Schematic of one-mass dynamic system.

According to the description of the SC principle, during gait, it is necessary that the knee impedance variation allows both body support and free movement of the leg. This dynamic requires high resistance at the movement, which can be defined using a system with force feedback. In this sense, admittance controllers are stable in high stiffness conditions; therefore, they are more suitable for implementation of an SC, due to the high and stable stiffness needed to avoid knee collapse during stance phase [34]. An admittance controller is one variation of impedance controller and its performance is determined by both the precision of force sensor and actuator position. Compared with impedance control, admittance behavior is often more easily implemented in hardware [35]. Thus, a proper measure of the effectiveness of a system, which is meant to produce a rapid motion response to external forces, is the mechanical admittance *Y* [36], defined as:

(1) Y=v/F,

where *v* is the velocity of the controlled system at the point of interaction, and *F* is the contact force at that point. A large admittance corresponds to a rapid velocity induced by applied forces. The dynamic behavior for the interaction between the actuator and the environment (in this case, the user during gait) can be expressed by the model shown in Figure 1.

In this model, the plant parameters are assumed to have values *M* and *D* for the mass and damping, respectively, in which an actuator exerts a force $F_{a}$, and the environment a force $F_{s}$. Then, the equation of motion for the system velocity is

(2) $$M\dot{v}+Dv=F_{a}+Fs.$$

$F_{a}$ and $F_{s}$ can be measured with a force sensor in order to obtain an interaction force *F*, hence it can be considered $F_{a}$ + $F_{s}$ = *F*.

In the Laplace domain, (1) can be expressed as

(3) $$v\left(s\right)=F{\left(Ms+D\right)}^{-1}.$$

For the implementation, the use of a velocity controller in the active knee orthosis is assumed. Based on Equations (1) and (3), the desired admittance can be expressed as:

(4) $$Y\left(s\right)={\left(Ms+D\right)}^{-1}.$$

The gain pattern to modulate the inertia and damping is applied to the relation of *M* and *D*, maintaining a ratio r=0.2 without considering units, where *r* was experimentally obtained here and expressed as:

(5) r=M/D,

with M>0 and D>0.

### 2.2. Knee Impedance Modulation

**Figure 2:**
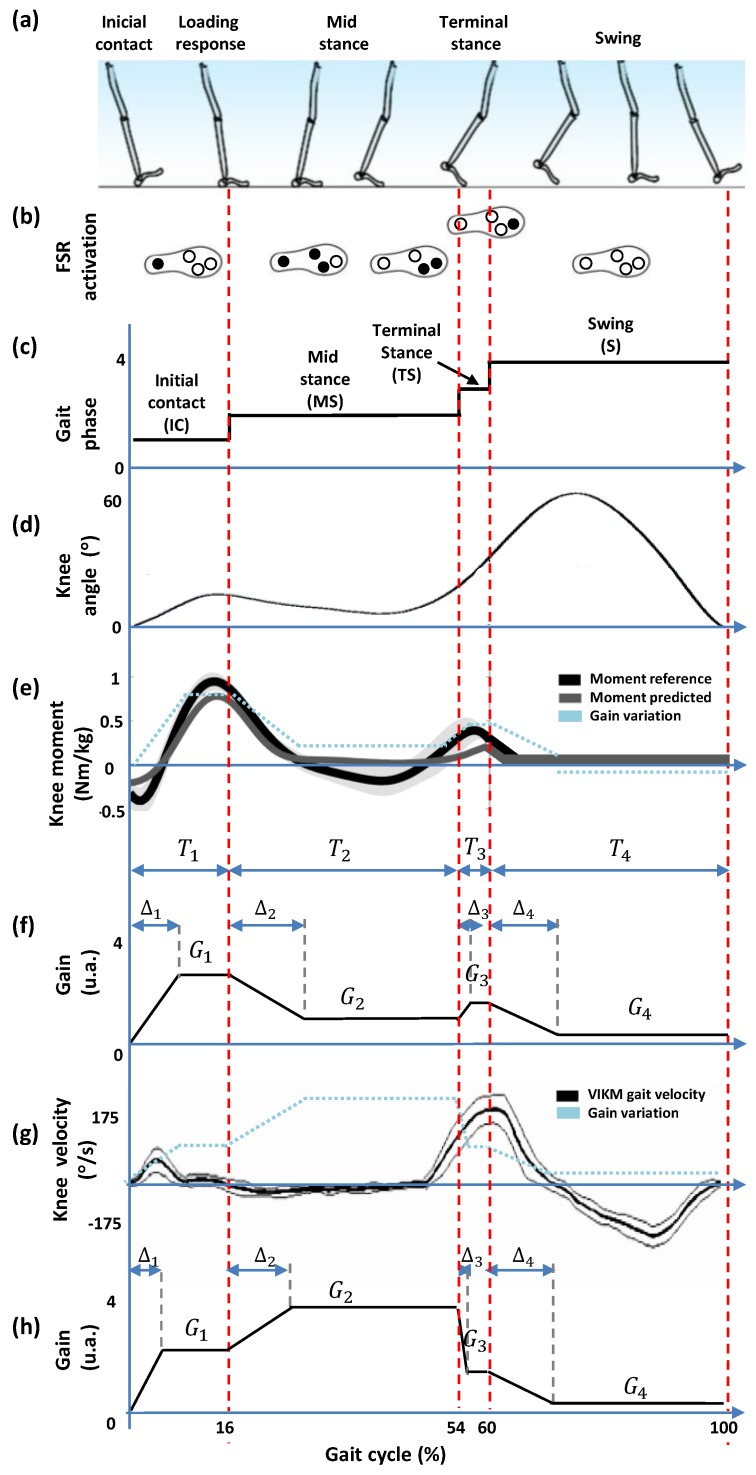
Events related to gait phases. (**a**) sub-phases of the gait cycle; (**b**) on-off sequence of force sensing resistors (FSR) throughout the gait cycle; (**c**) footswitch signal generated by the instrumented insole to identify gait phases; (**d**) knee angle throughout the gait cycle; (**e**) knee moment during gait, correspondent to the reference and predicted values of [21]; the gain variation was considered to define the gait pattern to knee impedance modulation during gait; (**f**) gain pattern $P_{1}$ based on the knee moment to decrease/increase gain values during gait phases for stance control; (**g**) knee velocity during gait using the variable impedance knee mechanism (VIKM) [38]; (**h**) gain pattern $P_{2}$ based on the the knee velocity to decrease/increase gain values during gait phases for a stance control.

In order to implement the SC control strategy with the admittance controller, a modulation through a variable gain *G* to increase or decrease the impedance components (damping and inertia) is required. The modulation must be according to the gait sub-phases to adapt the knee joint impedance during gait. Usually, most of the gait cycles are divided in the sequence of the following sub-phases [37]: (1) Initial contact (IC), defined by the heel contact; (2) Loading response and mid-stance (MS), defined by a flat foot contact; (3) Terminal stance (TS), defined by the heel off; (4) Swing (SW), defined by the foot off, as shown in Figure 2a.

This sequence offers information to develop an impedance modulation for an online variation of the knee joint impedance. The objective is to block the knee joint only in the stance phase to resist the knee flexion and allow free knee extension and free knee motion in the swing phase [25,39], in order to achieve, during gait, the knee angle, moment and velocity, as shown in Figure 2d–f.

For that, a different value of *G* for each sub-phase must be defined and vary smoothly. Figure 2f,h shows two examples of variation of *G* in a gait cycle. In both cases, the value for each sub-phase is $G_{1}$ for IC, $G_{2}$ for MS, $G_{3}$ for TS and $G_{4}$ for SW, which requires suitable times to increase/decrease *G* during the gait cycle, defined as: $\Delta_{t1}$, $\Delta_{t2}$, $\Delta_{t3}$ and $\Delta_{t4}$. Considering that the weight and the gait velocity are the two major parameters that affect the mechanical parameters of the knee [26], both weight and the gait velocity are considered here to define the corresponding *G* and $\Delta_{t}$.

The first example of variation of *G*, known as pattern 1 ($P_{1}$), shown in Figure 2f, corresponds to a pattern based on the knee moment variation shown in Figure 2e, which is the knee moment reported in a study of a model of a neuromuscular mechanism to regulate knee joint impedance during human locomotion [21]. Here, $P_{1}$ is adapted at the knee moment tendency throughout the gait sub-phases, in which $G_{1}$ has the highest values in the IC phase when the knee generates the first flexion. In sub-phase MS, $G_{2}$ decreases with a little increment in TS. The second example of variation of *G* shown in Figure 2h, termed pattern 2 ($P_{2}$), is a pattern obtained from a tendency marked in Figure 2g, which shows the knee velocity during walking using the variable impedance knee mechanism of an SC orthosis [38]. In this case, the highest value of $G_{2}$ is generated in the sub-phase MS, when the knee maintains the angle but the knee torque decreases. In both cases, an impedance modulation using $P_{1}$ and $P_{2}$ can generate a knee impedance that allows a shock damping during the weight acceptance stage (sub-phases IC and MS) where the knee applies a large moment.

For both patterns, the increase/decrease of *G* can be executed in times $\Delta_{1}$, $\Delta_{2}$, $\Delta_{3}$ and $\Delta_{4}$ for IC, MS, TS and SW, respectively. Hence, values of Δ depend on the period of duration of each sub-phase of the gait cycle. Then, considering *i* as the phase number assigned as follows: *i* = 1 for IC, *i* = 2 for MS, *i* = 3 for TS and *i* = 4 for SW, the duration of each sub-phase can be expressed as

(6) $$T_{i}=t_{GC}\left(Q_{i}/100\right)f_{s},$$

where $T_{i}$ is the duration of each sub-phase in seconds, $t_{GC}$ is the time of the gait cycle in seconds, $Q_{i}$ is the percentage of each phase with respect to the gait cycle, and $f_{s}$ is the sampling frequency in samples per second. As shown in Figure 2f, a suitable $\Delta_{i}$ does not have to exceed the corresponding $T_{i}$.

According to gait studies [40], $t_{GC}$ can be estimated through Equation 7:

(7) $$t_{GC}=SL/v_{u},$$

where SL is the stride length in meters, and $v_{u}$ is the user velocity in meters per second. SL can be estimated from the users height *H* in meters multiplied by the constant 0.826 [40]. Hence, $T_{i}$ can be expressed as

(8) $$T_{i}=0.826\left(HQ_{i}f_{s}\right)/100v_{u}.$$

Experimental tests to validate $Q_{i}$ with the instrumented insole were conducted, obtaining the following percentages for each phase: 16 ± 4%, 38 ± 6%, 6 ± 0.8% and 40 ± 4% for IC, MS, TS and SW phases, respectively. Based on the knee moment and velocity shown in Figure 2e,g, IC and MS are the more critical phases, which occur when a knee support is required. In this case, $\Delta_{t}$ should allow a time of stabilization in order to sustain the knee with a *G* constant for each phase.

**Figure 3:**
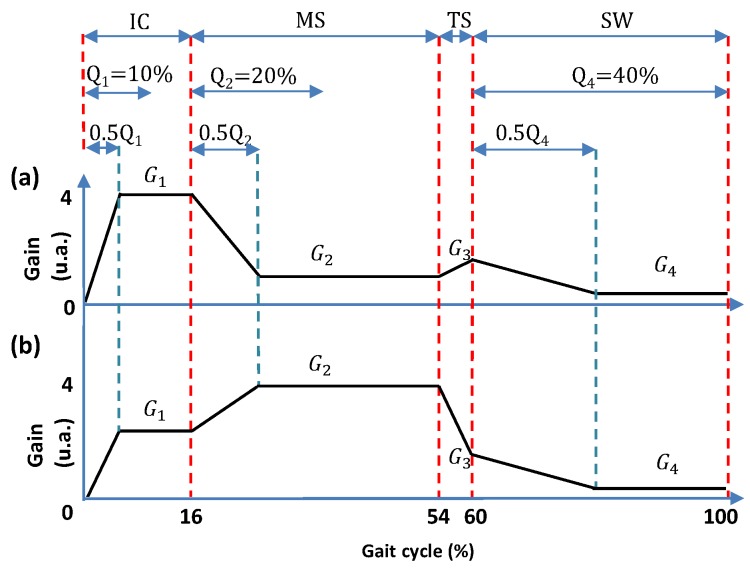
Percentage of each phase with respect to the gait cycle ($Q_{i}$) taken into account in this approach for (a) gain pattern $P_{1}$; (b) gain pattern $P_{2}$.

Therefore, for this method, values of $Q_{i}$ were defined as: $Q_{1}$ = 10%; $Q_{2}$ = 20% and $Q_{4}$ = 40%, as shown in Figure 3a,b. This consideration allows having a minimum period of time to increase or decrease the corresponding *G* and applies to patterns $P_{1}$ and $P_{2}$ (knee moment and velocity).

In relation to the sub-phase *TS*, it can be seen in Figure 3 that it has short duration with respect to other phases, and does not allow a suitable time for stabilization of *G*. For that reason, in order to simplify the method, 30% was chosen as the percentage for $Q_{3}$.

Considering that Δ represents 50% of its corresponding *T*, to obtain a smooth switching between the levels of *G*, Equation (9) is used:

(9) $$\Delta_{i}=\left(0.0413i/v_{u}\right)Hf_{s}.$$

**Figure 4:**
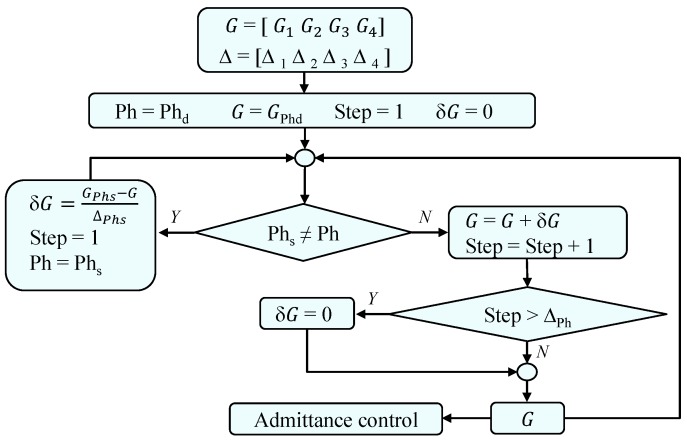
Flowchart of the algorithm used to generate the pattern *G*, where *Phs* is the output of the gait phase detector, and *Phd* is the default phase (recommended sub-phase mid-stance).

Figure 4 shows the flowchart of the algorithm implemented in Simulink/Matlab (2014b, The MathWorks Inc.) for online gain pattern generation, where *Phd* is the default phase from which the pattern *G* begins to be generated; *Phs* is the current phase recognized through the insole, and δ*G* is the gain increment for each phase.

Using the aforementioned gain patterns $P_{1}$ and $P_{2}$, the modulation of *M* and *D* in each gait sub-phase during the gait cycle can be expressed as

(10) $$M_{i}=M_{d}G_{i},$$

(11) $$D_{i}=D_{d}G_{i},$$

where $M_{d}$ and $D_{d}$ are the inertia and damping default values, respectively, according to Equation (5).

### 2.3. Gait Phase Detection

**Figure 5:**
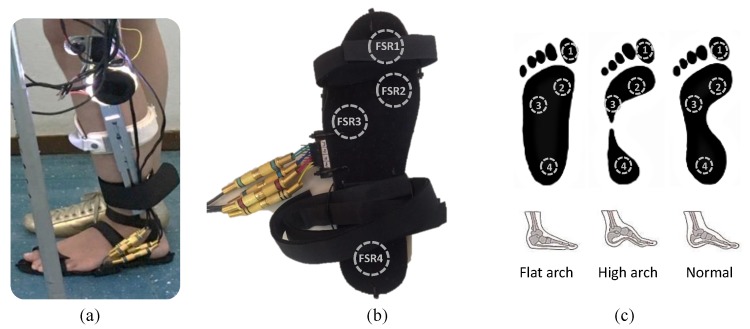
(**a**) instrumented insole implemented at the active knee orthosis; (**b**) FSR locations; (**c**) FSR locations at flat arch, high arch and normal foot.

The gait phase detection is required to implement the impedance modulation method proposed here, which is done by the instrumented insole built with force sensing resistors (FSRs) shown in Figure 5a. Four FSRs are placed on the plantar surface of the foot. Figure 5b shows the sensor locations, which are defined in the function of the peaks of the plantar pressure data reported in [41,42], corresponding to hallux bone (FSR1), 1st metatarsal (FSR2), 5th metatarsal (FSR3) and calcaneus (FSR4). These locations allow for acquiring more relevant ground reaction forces generated during gait to recognize stance sub-phases, which are suitable to use in feet with normal arch, high arch and flat foot Figure 5c.

The sensors employed are FlexiForce A401 (Tekscan Inc, Boston, MA, USA), which are force-sensing resistors with a sensing area of 25.4 mm and standard force range of 111 N. An electronic circuit was implemented to obtain output voltages proportional to the plantar pressure. To validate the insole data, a pressure sensitive gait mat GAITRite Electronic Walkway Platinum (CIR Systems Inc., Peekskill, NY, USA), 9 m long was employed. The signals of the insole were acquired with a DAQ USB-6009 (sampling frequency of 120 Hz) using the DAQ Express™ driver of National Instruments © (Austin, TX, USA) and Matlab software. The mat data were acquired at 1 kHz using the PKMAS (ProtoKinetics Movement Analysis) software (Franklin, NJ, USA) [43]. The acquisition data were synchronized throughout an external pulse. Two subjects (man: 35 years; 1.72 m; 70 kg and woman: 78 years, 1.75 m, 80 kg) walked at a comfortable velocity on the mat using the insole. Each subject completed six trials (each trial with six steps) completing 36 gait cycles. A concordance correlation analysis was performed to estimate the reliability of the insole pressure signals in relation to the foot pressure measured by the mat.Then, a gait phase detection algorithm based on a truth table from the combinations of the sensors during gait was programed in Matlab Simulink. The signals were acquired through an analog to digital acquisition card, model Diamond-MM-32DX-AT (32 inputs of 16 bits, 4 outputs of 12 bits, with maximum sampling frequency of 250 kHz) of a PC-104 computer, sampled at a frequency of 1 kHz, and conditioned through a low-pass filter Butterworth of 5th-order, with cutoff frequency of 10 Hz. Afterwards, the signals were compared to a threshold of 0.5 V in order to obtain contact information (on-off) from the footswitch. In order to recognize the gait sub-phases IC, MS, TS and SW, the combinations shown in Figure 2b were considered. Then, a truth table implemented in Simulink/Matlab, which includes these combinations, was used to obtain a logic scheme to generate the footswitch signal shown in Figure 2c.

### 2.4. Active Knee Orthosis

**Figure 6:**
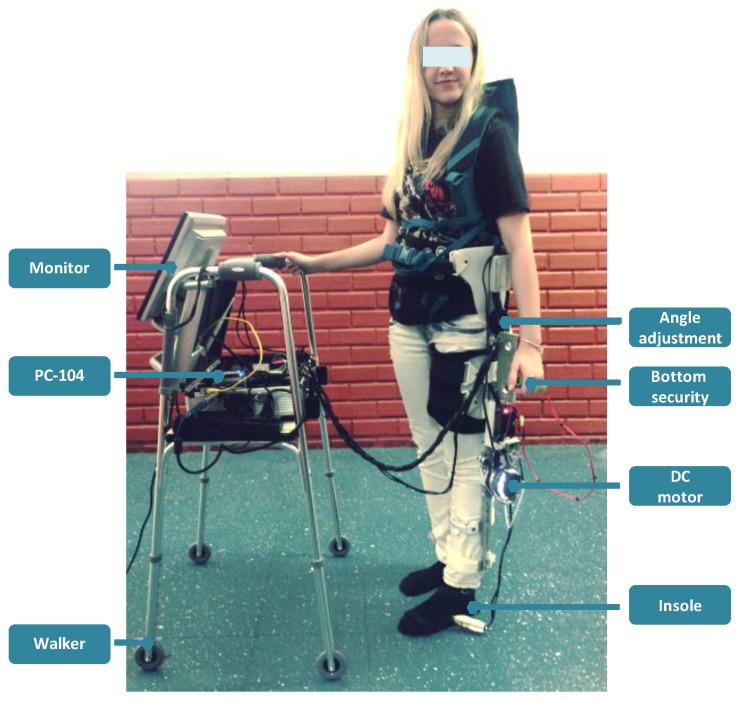
Advance Lower Limb Orthosis for Rehabilitation (ALLOR) built for this research.

Figure 6 shows the active knee orthosis developed at the Federal University of Espirito Santo (UFES/Brazil) known as ALLOR (Advance Lower Limb Orthosis for Rehabilitation), which was used to test the admittance controller with the modulation method proposed here.

ALLOR is a two degree of freedom orthosis composed of an active knee joint and a passive hip, which moves in the sagittal plane during the walking. The hip joint has a manual flexion and extension angle regulator from 0 to 80 degrees. Although this joint is not active, the regulation, according to the user requirements, allows for establishing a safe range of motion. During gait, the physiological range of motion (flexion and extension) must be adjusted to $\pm 20^{\circ }$ for hip, while the movements of the frontal plane are restricted. ALLOR is mounted on the left leg of the user with the axis of rotation of the orthosis joint aligned with the axis of the user knee and hip joints. To ensure a correct alignment during operation, a backpack and rigid braces at the thigh and shank with velcro straps are used. ALLOR weighs 3.4 kg (including 0.8 kg of the backpack) and is adaptable to different anthropometric setups, which include heights of 1.5 to 1.85 m and weights from 50 to 95 kg. It provides both mechanical power to the knee joint and feedback information related to knee angle, interaction torque and gait phases. It was developed for knee rehabilitation in both sit position and during gait. In this last case, the user must use the walker shown in Figure 6.

The components of the active knee joint are a brushless flat motor (model 408057), a Harmonic Drive gearbox (model CSD-20-160-2A-GR) and an analog pulse-width modulation (PWM) servo drive (model AZBH12A8). Additionally, ALLOR is equipped with a strain gauge arrangement (Wheatstone bridge configuration), which measures the torque produced by its interaction with the user. A precision potentiometer model 157S103MX) is used as an angular position sensor to measure knee angles. ALLOR also uses Hall Effect sensors inside the motor to compute angular speeds of the actuator. The computer used to implement the control software is a PC/104, which is a standard for embebbed computers, in which the architecture is built by adding interconnected modules through an industry standard architecture (ISA) data bus. The modules are a motherboard, power source, ethernet communication and an analog to digital (A/D) acquisition card, model Diamond-MM-32DX-AT (32 inputs of 16 bits, four outputs of 12 bits, with maximum sampling frequency of 250 kHz). All sensors, acquisition and velocity driver are connected through the A/D card. The whole system requires 24 V/12A DC power supply and uses a controller area network (CAN) bus running at 1 Mbps. The control software was developed in Simulink/Matlab and uses a real-time target library. Safety conditions are incorporated at the ALLOR control system along with mechanical stops, which ensure that the actuator operates within the normal range of motion of the knee, allowing safe use.

**Figure 7:**
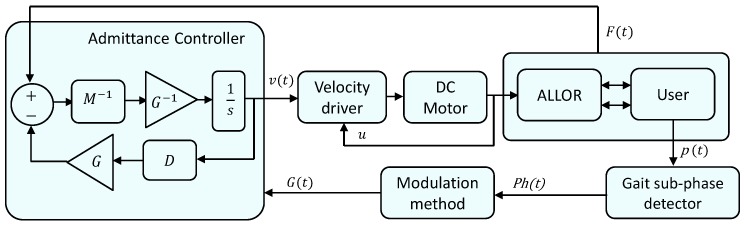
Admittance controller implemented at the active knee orthosis.

Figure 7 shows the admittance controller implemented in Simulink/Matlab, which is based on Equation (2). *Ph(t)* is the phase, which is recognized online by the gait phase detector through the use of the instrumented insole. *G(t)* is a variable gain for the impedance modulation. The controller also includes an outer force control loop implemented over a inner velocity control loop, in which the motor controller performs the velocity closed-loop control with information feed from Hall sensors on the motor structure. In this controller, the axis of the subject knee joint is considered to be aligned to the axis of the knee joint of the active orthosis.

### 2.5. Experimental Protocol

In order to evaluate the proposed method, the following protocol was conducted with ALLOR. Three healthy subjects, female (26 ± 5.13 years; height 1.62 ± 0.03 m; weight 56 ± 8.75 kg) without lower-limb injury or locomotion deficits, participated in the tests. Written, informed consent was obtained from each subject before participation. The Ethics Committee of the Federal University of Espirito Santo approved this protocol, with number: 64801316.5.0000.5542. At the beginning of the test, the subjects were asked to perform a trial with the walker and without ALLOR, walking a distance of 10 m at a comfortable speed for each one. Then, the gait velocity was calculated to obtain the *v* reference value needed to adjust *G*. Then, ALLOR was mounted on the subject to perform three level-ground walking trials in a distance of 10 m with the following patterns for *G*: (1) knee moment based-pattern shown in Figure 3a, termed $P_{1}$, with $G_{1}=0.7$ W, $G_{2}=0.2$ W, $G_{3}=0.3$ W and $G_{4}=0.1$ W; (2) knee velocity based-pattern shown in Figure 3b, termed $P_{2}$, with $G_{1}=0.4$ W, $G_{2}=0.7$ W, $G_{3}=0.2$ W and $G_{4}=0.1$ W; (3) pattern termed $P_{3}$ to perform a gait without knee modulation, maintaining $G_{4}$ corresponding to SW phase in all the gait cycle, hence $G_{1}$ = $G_{2}$ = $G_{3}$ = $G_{4}$ = 0.1 W, where *W* is the user’s weight.

For the three patterns of *G*, the impedance parameters *M* and *D* are set as 0.5 kg and 2.5 N/(m/s), respectively, which are obtained experimentally from gait tests with ALLOR. The trials were carried out at slow speed, determined by the subject, and were performed with the acquisition hardware attached to a four wheel walker as shown in Figure 6, in order to have a mobile platform during the tests. Each trial had an average of seven steps, and three trials with each pattern *G* were performed. The patterns ($P_{1}$, $P_{2}$ and $P_{3}$) were randomly applied at the controller in order to not influence their perception regarding the effects introduced by each modulation pattern.

**Figure 8:**
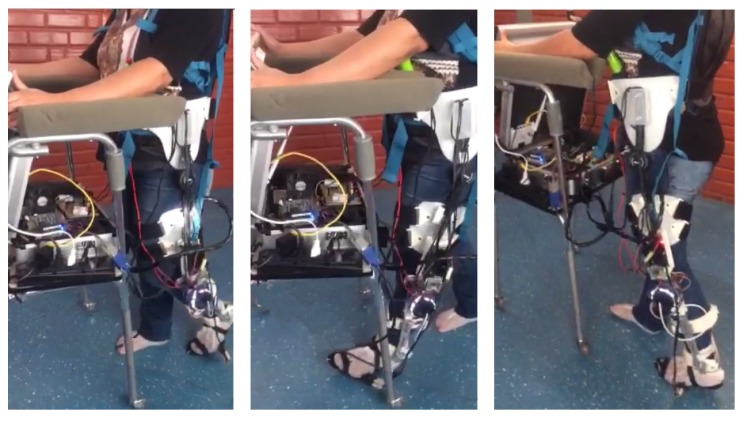
Sequence of an experiment conducted at 0.2 m/s by a subject wearing ALLOR with the stance control using the knee impedance modulation based on the knee moment during gait.

During these experiments, the subjects were asked to accomplish their normal gait patterns, considering the imposed system (ALLOR and walker) and a slow speed. The use of a walker in this study was with the goal of emulating the same conditions of patients or subjects with disabilities, which will need the walker to improve their stability and ambulatory ability themselves in order to feel safety during gait. A sequence of a subject performing this protocol is illustrated in Figure 8.

### 2.6. Statistical and User’s Satisfaction Analysis

For statistical analysis, data from the three subjects that participated in the test, related to speed of walking, cadence, stance phase percent and maximum knee flexion in swing phase, were used. Friedman test (non-parametric statistical test) was used to compare the three gain modulation patterns. The level of significance was set at *p* < 0.05.

Finally, a survey to measure satisfaction with the use of assistive technology, the adapted Quebec User Evaluation of Satisfaction with Assistive Technology (QUEST 2.0) was used [44]. QUEST 2.0 may be used to evaluate the user satisfaction through 12 questions separated in two items: assistive technology and services. In this study, only the issues related to assistive technology (dimensions, weight, adjustments, safety, durability, simplicity of use, comfort, and effectiveness) were evaluated, since it is a non-commercial product in the phase of controlled tests. The score for each question ranges from 1 to 5 ( 1 “not satisfied at all”; 2 “not very satisfied”; 3 “more or less satisfied”; 4 “quite satisfied”; and 5 “very satisfied”), and, finally, an average score is taken for the number of valid questions answered. The subjects were asked to select the three most important items.

## 3. Results

### 3.1. Instrumented Insole

**Figure 9:**
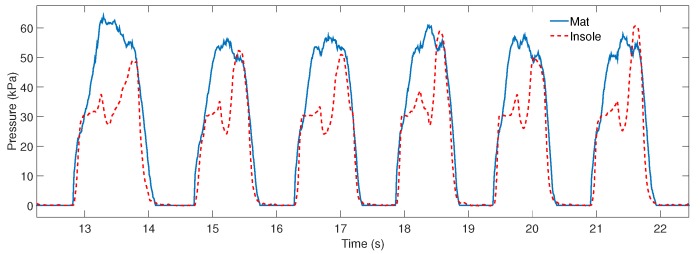
Mat and insole plantar pressure data during six steps.

Figure 9 shows the mat and insole pressure data during the test with subject 1. For both subjects, 144 steps were collected and the pressure data of the insole presented acceptable values of precision (r>0.92 ± 0.02) with respect to the mat pressure, which has an accuracy of (Cb>0.82 ± 0.02), and acceptable reproducibility (pc>0.89 ± 0.03).

Although the pressure data presented differences in the first sub-phases of stance phase, as shown in Figure 9, the insole showed a good response to recognize stance and swing phase. Hence, the algorithm used in this research to determine the gait phases uses data from the insole.

### 3.2. Knee Impedance Modulation

**Figure 10:**
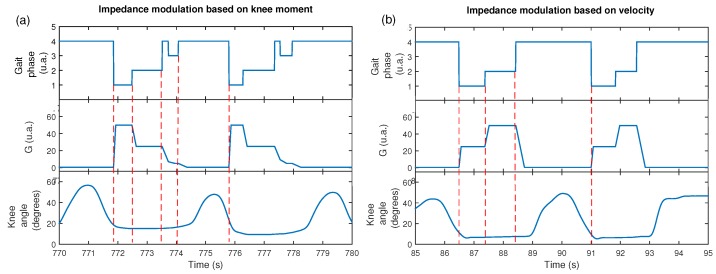
Footswitch signal, gain variation and knee angle during gait with impedance modulation. (**a**) modulation obtained from the pattern $P_{1}$ based on normal velocity; (**b**) modulation obtained from the pattern $P_{2}$ based on the knee moment.

Two gain patterns for knee impedance modulation were used to control the knee orthosis: the first based on the knee moment during gait $P_{1}$, the second based on knee velocity $P_{2}$. For purposes of comparison with both knee modulation patterns, a third pattern $P_{3}$ was also used to develop a free gait without knee impedance modulation. Results are shown in Figure 10 to demonstrate the efficiency of the knee modulation proposed for SC assistance, where it is possible to see the variation of the knee angle during gait using patterns $P_{1}$ and $P_{2}$. Here, the variation of the knee angle in time shows that the subject walked approximately with the same velocity. The knee angle showed similar amplitudes for both patterns. Even though the footswitch signals presented a false negative in sub-phase TS as shown in Figure 10a, the method may adapt the modulation of the gain *G* in this period and maintain the expected value of *G*.

**Figure 11:**
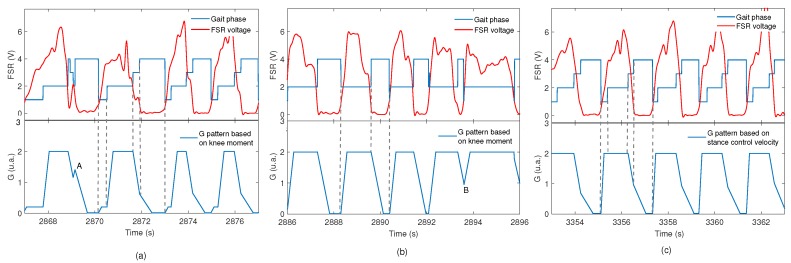
*M* variation during gait cycle. (**a**) variation of M during a gait test, which generates four sub-phases: initial contact (IC), mid-stance (MS), terminal stance (TS) and swing (SW); (**b**) example with three sub-phases; (**c**) example that shows the variation of M during a gait cycle with noise.

For the pattern *G* based on velocity $P_{2}$, Figure 11a–c shows the variation of *G* during the gait, which generates different footswitch signals at different walking speeds.

The footswitch signal of the instrumented insole showed good performance to measure the four gait sub-phases considered for the impedance modulation. The gait pattern also was different in some cases, as shown in Figure 10, which is considered common due to the gait dynamic. These examples demonstrate that a specific subject does not present a single characteristic gait cycle. It is reported in literature that the percentage of atypical cycles in the healthy adults is from 1% to 3% [29]. Despite this, the modulation method proposed here was able to generate pattern *G* to obtain an SC performance even with a non ideal footswitch signal, as shown in Figure 11.

**Figure 12:**
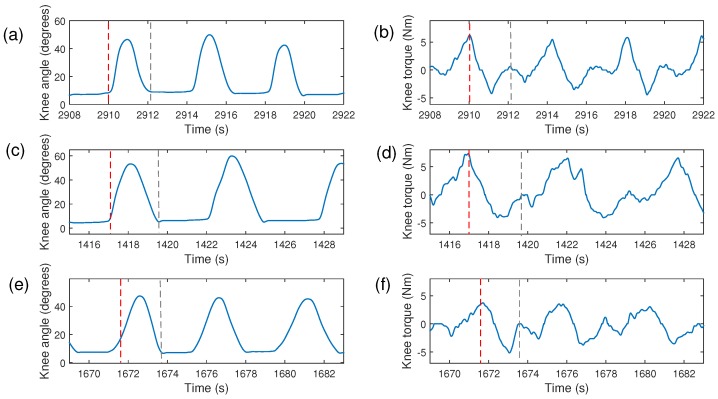
Knee angle and knee torque during knee impedance modulation with patterns *G*: $P_{1}$ (**a**,**b**); $P_{2}$ (**c**,**d**); $P_{3}$ (**e**,**f**).

Figure 12 shows the knee torque and knee angle of subject 1, with impedance modulation patterns $P_{1}$, $P_{2}$, and $P_{3}$ during the gait. The maximum torque is presented at the beginning of the knee flexion (marked with red dashed line) for $P_{1}$ and $P_{2}$. Another increment of knee torque was obtained at the beginning of the stance phase (marked with gray dashed line) for the three patterns.

In [19], a gait analysis with an active knee orthosis without a walker was conducted, in which the torque with a position control at 0.28 m/s was approximately ±5 Nm. In addition, this study reported that the torque with an adaptive impedance control at 0.28 m/s and 0.44 m/s may have values of ±10 Nm. In our work, walking with ALLOR and the walker at 0.2 m/s implies an interaction torque of ±5 Nm, as shown in Figure 12. In this sense, the new method for knee impedance modulation proposed here presents less knee torque than the torque presented in [19], with both position control and knee impedance modulation. Hence, the method based on FSR sensors for gait phase segmentation may be used to modulate knee impedance without demanding additional knee torque from the user during walking.

Table 1 shows the temporospatial and kinematic information of the subjects when walking using the three *G* patterns $P_{1},P_{2}$ and $P_{3}$.

For the three subjects, the results shown in Table 1 demonstrate no significant difference in gait velocity (p=0.067), percentage of ST phase of the gait cycle (p=0.44) and maximum flexion in SW (p=0.153) between $P_{1}$, $P_{2}$ and $P_{3}$. However, a significant difference in walking with $P_{2}$ resulted in a slower cadence in 16% and 10% compared with $P_{1}$ and $P_{3}$, respectively (p=0.0032).

Considering both modulation patterns, pattern *G* that presents better temporospatial parameters was the knee moment based on pattern $P_{1}$, which reported highest walking speed, cadence and stance phase percentage of the gait cycle compared with $P_{2}$. However, regarding the kinematics, the maximum knee flexion was increased (39.92 ± 12.40${}^{\circ }$) using the pattern $P_{2}$ based on knee velocity.

In addition, the duration of the swing phase during walking generally represented 50% to 56% of the gait cycle, as shown in Table 1. A study that describes gait analysis using an exoskeleton with walker [45] reports a swing phase around 37% of the gait cycle with healthy subjects. On the other hand, in [46] , a gait analysis with an hybrid neuroprosthesis for SC is performed, which reports that the swing phase during evaluation with nondisabled subjects represents 36% to 51% of the gait cycle. Then, the swing phase percentage obtained during gait with the proposed method agrees with a gait analysis that considers SC. Furthermore, a study of gait analysis with assistive devices tested with pathological cases, such as spinal cord injury [46], reports that the swing phase represents 25% of the gait cycle. In [47], other research of a gait analysis using a knee-ankle-foot orthoses with a powered knee joint is reported, whose swing phase during evaluation with poliomyelitis subjects represents 36% to 51% of the gait cycle. Then, in this sense, an important future task is to analyze the real-time adjustment of knee impedance in pathological gait.

Walker assisted gait with healthy subjects has been reported values between 0.17 m/s and 0.29 m/s depending on the body weight bearing patterns of the leg [48]. In [49], a gait assisted by a smart walker without orthosis in post-stroke subjects showed gait velocity values between 0.23 m/s and 0.44 m/s. Then, for the three patterns used here, the walking velocity is within that range of the first case, which means that the effect of ALLOR with knee impedance modulation does not produce a significant speed reduction in walker-assisted gait. In addition, this value indicates that the incorporation of a smart walker can be considered for test with post-stroke subjects.

Regarding the maximum knee flexion during swing phase, the three patterns present similar values, agreeing with [45], where the walking speed using a powered gait orthosis with a walker has been reported as being 48 ± 10${}^{\circ }$. Here, it should be made clear that, during walker assisted gait, the gait velocity and knee flexion in phase SW are lower than a normal gait.

Regarding the QUEST survey, the user satisfaction with ALLOR controlled by the proposed approach was scored as: dimensions: 4.00 ± 0.00, weight: 3.67 ± 0.58, adjustment: 3.33 ± 0.58, safety: 4.00 ± 0.00, durability: 3.00 ± 1.00, ease of use: 3.33 ± 1.15, comfort: 3.33 ± 0.58 and effectiveness: 3.33 ± 0.58, in a range of 0 to 5.

Based on the experience after and during this study, it was verified that the use of our system requires a therapist or assistant to mount the orthosis on the user. The total time required for this task is approximately 8 min with subjects familiarized with ALLOR. When it is being used for the first time, more minutes are required, in order to adjust the length of leg and thigh segments along with hip angle adjustment. In this case, the total amount of time is from be 20 to 25 min.

## 4. Discussion

This work evaluated the effect on walking with an active knee orthosis (ALLOR) while using two knee impedance modulation patterns: $P_{1}$ (based on knee moment) and $P_{2}$ (based on knee velocity), which incorporates an SC strategy. The main functional purpose of the SC strategy is to provide free movements in swing phase and provide a support to knee joints during stance phase.

The three patterns present no significant difference in walking speed, stance phase percentage of the gait cycle and maximum flexion during swing phase, as indicated in Table 1. The results of our study demonstrated that the proposed patterns $P_{1}$ and $P_{2}$ could be used to improve knee support in stance phase. Hence, both gain patterns are suitable to modulate the knee impedance and assist the knee joint under the SC strategy using an admittance controller.

The variation of the knee impedance was performed considering two implications for the design of stance control orthoses: walking speed and weight. In fact, literature shows that the following parameters: stiffness, knee flexion and extension, and maximum moment change with gait speed [26]. On the other hand, it is also reported that the stiffness of the parallel assistive device should be modified as the load or pilot weight changes [26]. In this sense, both patterns *G* change the impedance at knee during gait cycle, increasing or decreasing it, according to the both weight and velocity of the subject. Then, the proposed approach may also be considered to evaluate knee impedance variation to design efficient assistive devices. The weight percentage considered for each gait phase was due to the characteristics of the subjects during tests, which are healthy adults that have normal gait development. For this study, gait assisted by a walker was chosen due to it allowing offering safety to the users. It is worth mentioning that parallel bars, crutches or canes can be also used as support elements instead of the walker. For future tests with subjects with disabilities, the total weight of the user will be considered, according to recommendations for the design of parallel assistive devices [26].

In addition, for gait phase recognition in pathological cases, such as stroke survivors with foot drop problems, which present different footswitch signals (percentage of atypical cycles from 11% to 100% in pathological subjects) [29], an alternative for gait phase detection might be necessary. Therefore, an individual study to define it is recommended to design an insole with additional sensors or programming a gait-phase detector for each case must be conducted. In addition, data fusion techniques may be used, taking into account the knee angles acquired by goniometers or inertial measurement units (IMUs). Based on the experience and participants’ comments, the instrumented insole was comfortable to use. In future works, the insole will be used to study plantar pressure in order to detect alterations in gait, and allow comparing stroke survivors with healthy people. Stroke survivors usually adopt walking strategies, such as heel walking, planar stride or low heel pressure. These gait alterations can evolve to more complex musculoskeletal disorders, which influence functional activities. Plantar pressure can inform about these alterations, calculating the gait variability over time [31], and therapists can use this data as feedback to help strategies for rehabilitation avoid the evolution of gait disorders.

In relation to user satisfaction, results show that the lowest score (3.00) was related to “durability”, while questions on “adjustment”, “ease of use”, “comfort” and “effectiveness” received a mean value (3.33) on the QUEST score. In this sense, some hardware adjustments are needed to obtain a more robust system and improve the “adjustment”, “ease of use” and “comfort” items, such as new materials to adjust the exoskeleton and to decrease structure and the hardware weight. The comfort is associated with adjustment of actuators and biomechanics of human movement. Some factors such as sensors, straps and weight affect the gait of healthy people, causing more energy costs [15]. Physiological theories have been developed to address these limitations in wearable robots [50], but more clinical trials are necessary to determine how these adjustments influence normal and pathological gaits, making these exoskeletons more easy to use in daily activities. It is worth noting that participants in this study this system for the first time. Based on their comments after the experimental protocol, the time required to adjust the device will be improved. We considered that offering unilateral knee assistance for healthy subjects can influence this discomfort.

Regarding the “effectiveness”, a clinical protocol with a therapist is needed to address this issue in practice, in order to evaluate ALLOR with knee impedance modulation and its effect on patients. With this purpose, a graphical user interface will be adapted for the therapist who accompanies the rehabilitation, in order to facilitate the programming and monitoring of variables, such as: knee angle and torque, plantar pressure, number of steps and choosing pattern *G* for knee impedance modulation.

To conclude, our control method constitutes an approach to assist knee movement in stance phase. Future works will focus on implementing a position controller for swing phase or functional electrical stimulation FES, in order to apply force to the advance leg. In addition, future efforts will investigate correlations between the FSR activation and the knee joint impedance during walking on treadmills and stair climbing.

## Figures and Tables

**Table 1:** Mean and standard deviation for temporospatial parameters, and maximum flexion during swing phase for subjects wearing the active knee orthosis controlled by the stance control strategy using three types of impedance modulation patterns.

	Gait Velocity (m/s)	Cadence (steps/min)	Stance Phase (%gait cycle)	Maximum Flexion in Swing Phase (${}^{\circ }$)
P1	0.18 (0.07)	26.76 (6.87)	49.73 (7.87)	36.44 (9.56)
P2	0.14 (0.05)	22.41 (3.25)	46.19 (9.04)	39.92 (12.40)
P3	0.18 (0.04)	24.02 (6.22)	44.51 (7.75)	39.0 (10.61)
*p*-value	0.0670	0.0032 *	0.4493	0.1534

P1, Gain pattern based on knee moment; P2, Gain pattern based on knee velocity; P3, Gain pattern for gait without knee support in the stance phase. * significant difference

## Abbreviations

The following abbreviations are used in this manuscript:

ALLOR	Advance Lower Limb Orthosis for Rehabilitation
*D*	Damping
*F*	Force
FSR	Force sensing resistor
*G*	Gain for impedance modulation
*H*	ALLOR user’s height
IC	Initial contact phase
*M*	Mass
*p*	Plantar pressure
$P_{1}$	Gain pattern for impedance modulation based on knee moment during gait
$P_{2}$	Gain pattern for impedance modulation based on knee velocity during gait
$P_{3}$	Gain pattern for impedance modulation to obtain a free movement
Ph	Phase
*Q*	Percentage
QUEST	Quebec User Evaluation of Satisfaction with Assistive Technology
SC	Stance control
ST	Stance phase
SW	Swing phase
*t*	Time
*W*	ALLOR user’s weight
